# Uniform Na^+^ Doping‐Induced Defects in Li‐ and Mn‐Rich Cathodes for High‐Performance Lithium‐Ion Batteries

**DOI:** 10.1002/advs.201802114

**Published:** 2019-05-17

**Authors:** Wei He, Pengfei Liu, Baihua Qu, Zhiming Zheng, Hongfei Zheng, Pan Deng, Pei Li, Shengyang Li, Hui Huang, Laisen Wang, Qingshui Xie, Dong‐Liang Peng

**Affiliations:** ^1^ Department of Materials Science and Engineering State Key Lab of Physical Chemistry of Solid Surface Collaborative Innovation Center of Chemistry for Energy Materials College of Materials and Pen‐Tung Sah Institute of Micro‐Nano Science and Technology Xiamen University Xiamen 361005 P. R. China

**Keywords:** corrosion effect, doping, Li‐ and Mn‐rich cathodes, lithium‐ion batteries, stacking faults

## Abstract

The corrosion of Li‐ and Mn‐rich (LMR) electrode materials occurring at the solid–liquid interface will lead to extra electrolyte consumption and transition metal ions dissolution, causing rapid voltage decay, capacity fading, and detrimental structure transformation. Herein, a novel strategy is introduced to suppress this corrosion by designing an Na^+^‐doped LMR (Li_1.2_Ni_0.13_Co_0.13_Mn_0.54_O_2_) with abundant stacking faults, using sodium dodecyl sulfate as surfactant to ensure the uniform distribution of Na^+^ in deep grain lattices—not just surface‐gathering or partially coated. The defective structure and deep distribution of Na^+^ are verified by Raman spectrum and high‐resolution transmission electron microscopy of the as‐prepared electrodes before and after 200 cycles. As a result, the modified LMR material shows a high reversible discharge specific capacity of 221.5 mAh g^−1^ at 0.5C rate (1C = 200 mA g^−1^) after 200 cycles, and the capacity retention is as high as 93.1% which is better than that of pristine‐LMR (64.8%). This design of Na^+^ is uniformly doped and the resultanting induced defective structure provides an effective strategy to enhance electrochemical performance which should be extended to prepare other advanced cathodes for high performance lithium‐ion batteries.

## 1 Introduction

To meet the rapidly growing demand in electric vehicles (EVs), cathodes of lithium‐ion batteries (LIBs) with high energy density will be more urgently needed than ever before.^1^, ^2^, ^3^, ^4^ In response to this demand, enormous time and efforts have been invested in searching for satisficing solutions to achieve the goals of low cost, long cycle life, high specific capacity and high safety. In this context, Li‐ and Mn‐rich (LMR) transition metal (TM) oxides are labeled as one of the most promising candidates for next generation cathode materials of rechargeable LIBs because of their prominent energy density of ≈1000 Wh kg^−1^.^5^, ^6^ Despite the attraction in energy density, there are still many problems need to be solved before their commercialization.^7^, ^8^, ^9^, ^10^ For instance, voltage decay and capacity fading resulted from structural degradation and phase transition are major obstacles for nearly all LMR cathodes with chemical formula *x*Li_2_MnO_3_·(1−*x*)LiTMO_2_ (transition metal (TM) = Ni, Co, Mn, etc.).^11^, ^12^, ^13^, ^14^, ^15^, ^16^

Doping is considered as an effective strategy to improve the performance of the LMR cathode materials.^17^ Alkali metal elements like K,^18^, ^19^, ^20^ Na,^21^, ^22^ alk‐earth metal elements Mg^23^, ^24^, ^25^, ^26^ and transition elements including Fe,^27^, ^28^ Cr,^29^ Ti,^30^ and La,^31^ had been used to optimize the ionic diffusion coefficient and enhance the structure stability. However, most of the conventional doping strategies would induce the formation of a thin coating‐layer on the surface because of their larger ionic radii in comparison to lithium; thus, the doped elements were aggregated at the surface instead of inside.^32^ For instance, Li et al.^33^ have clearly elucidated that the mechanisms of surface‐dopant‐coated layer in stabilizing particles was due to the enhancement of TM—O bond strength and the alleviation of oxygen loss. Although these surface modification methods work effectively in promoting ionic diffusion coefficient and stabilizing cathode–electrolyte interface, the internal cathode particles in a deep charged state also need to be protected.^34^, ^35^, ^36^ Furthermore, the nano‐sized coating layers are usually easy to crack during prolonged cycles, and the electrolyte would directly penetrate through the ruptured‐surface of secondary particles and consequently dissolve the inner pristine‐LMRs, resulting in enormous loss of TM ions and potential safety risks.^8^, ^37^ Recently, Yan et al.^37^ have verified that infusing a solid electrolyte (Li_3_PO_4_) into the grain boundaries of primary particles could dramatically enhance the capacity retention and voltage stability while the surface coating did not work. This approach not only prevented the cracking and phase transition, but also improved the interfacial stability, enabling an excellent cycle stability. Therefore, this work provides us with an idea for designing Na^+^ deep doping in LMR cathodes uniformly to avoid the formation of cracks, maintain the integrity of electrode particles during prolonged cycles, and utilize the Na^+^'s own intrinsically high bond strength with oxygen framework to inhibit the oxygen loss and preserve the layered structure.^16^, ^33^ Further, the abundant defects like stacking faults induced by the micro‐strain resulted from doping lie into the bulk materials to deliver an enhancement for electrochemical performance.^38^

In this work, we introduce abundant stacking faults in the as‐prepared Na^+^‐doped LMR to promote its electrochemical properties. Also, the surfactant (sodium dodecyl sulfate, SDS) that was added during the preparation process ensures a deep and uniform doping of Na^+^ not just gathering on the surface. In addition, the pristine electrode materials and after 200 cycles at 0.5C rate are systematically investigated by the XRD refinement, HRTEM, and Raman spectrum to show the protective effect of Na^+^ doping and the performance improvement by defects. As expected, the Na^+^ ions distribute uniformly inside the layered‐lattices for dozens of nanometers with the assistant of surfactant, which can suppress the depletion of TM ions in electrolyte and phase transition. The corresponding discharge specific capacity of the modified LMR material retains 221.5 mAh g^−1^ after 200 cycles at 0.5C rate (1C = 200 mA g^−1^), and the capacity retention is as high as 93.1%, which are better than 139 mAh g^−1^ and 64% for pristine‐LMR. Therefore, the results above reveal that these formed abundant nano‐defects existing in the SDS‐assisted uniform Na^+^‐doped LMR play an important role in significantly improving electrochemical performance. Compared with the conventional doping processes, the SDS‐assisted Na^+^‐doping method demonstrates an effective inhibition of the corrosion from electrolyte, delivering a decent protection to the electrode particles. Also, the induced defective structure is found valid for achieving the enhanced electrochemical performance.

## 2 Results and Discussion

A simple co‐precipitation method and the subsequent high‐temperature calcination process were used for the synthesis of LMRs (see Section 5 for more experimental details). The optimal calcination temperature is determined to be 800 °C to ensure a desirable electrochemical performance (Figure S1, Supporting Information). In order to uncover the effects of SDS and Na^+^‐doping, three experiments with same external conditions were set up. The corresponding cathode materials were fabricated using undoped, Na^+^‐doped and SDS‐assisted Na^+^‐doped particles, and denoted as pristine‐LMR, Na‐LMR, and Na/SDS‐LMR for convenience. The corresponding carbonate precursors were denoted as pristine‐NCMCO, Na‐NCMCO, and Na/SDS‐NCMCO, respectively.

SDS is a typical anionic surfactant with general amphipathic nature, which might be an important reason for inhibiting the formation of surface coating layer.^39^ To reveal the mechanism, we must first confirm that SDS still presents in solid materials rather than only existing in solution system. The Fourier transform infrared spectroscopy (FTIR) was used to identify the existence of SDS in carbonate precursors (Figure S2, Supporting Information). The TG curves in Figure S3, Supporting Information, demonstrate that there are three stages (separated by green vertical dot lines) of mass loss for pristine‐NCMCO and Na‐NCMCO, but four stages for Na/SDS‐NCMCO in Figure S3c, Supporting Information. It is reasonable to speculate that the extra stage may relate to the decomposition of SDS (melting point of 204–207 °C). The first mass loss that appears below 181.9 °C is mostly due to the removal of absorbed water.^16^ The subsequent mass loss emerged between 181.9 and 389.6 °C would be attributed to the decomposition of TM‐CO_3_ (NCMCO) and formation of TM‐O (NCMO); the endothermic peak presented at 347 °C agrees well with the decomposition temperature of MnCO_3_ (347 °C). The third stage between 390 and 630 °C is probably ascribed to the evolution of TM‐Os and the transition of crystal‐oxide phases.^40^ Obviously, the excrescent endothermic peak existed in the TG curve of Na/SDS‐NCMCO between 200 and 296.9 °C reveals the weight fraction of SDS, which is identified as 5.04% from the summarized data in **Table** **1**.

**Table 1:** Summary of the mass loss in different temperature range by thermogravimetric analysis

Sample [%]	Pristine‐LMR	Na‐LMR	Na/SDS‐LMR	M‐SDS [%]
MI	7.54	9.53	5.31	5.04
MII	18.98	18.98	24.02	
MIII	7.98	8.15	7.02	
Total mass loss [%]	34.50	36.66	36.35	

**Figure 1:**
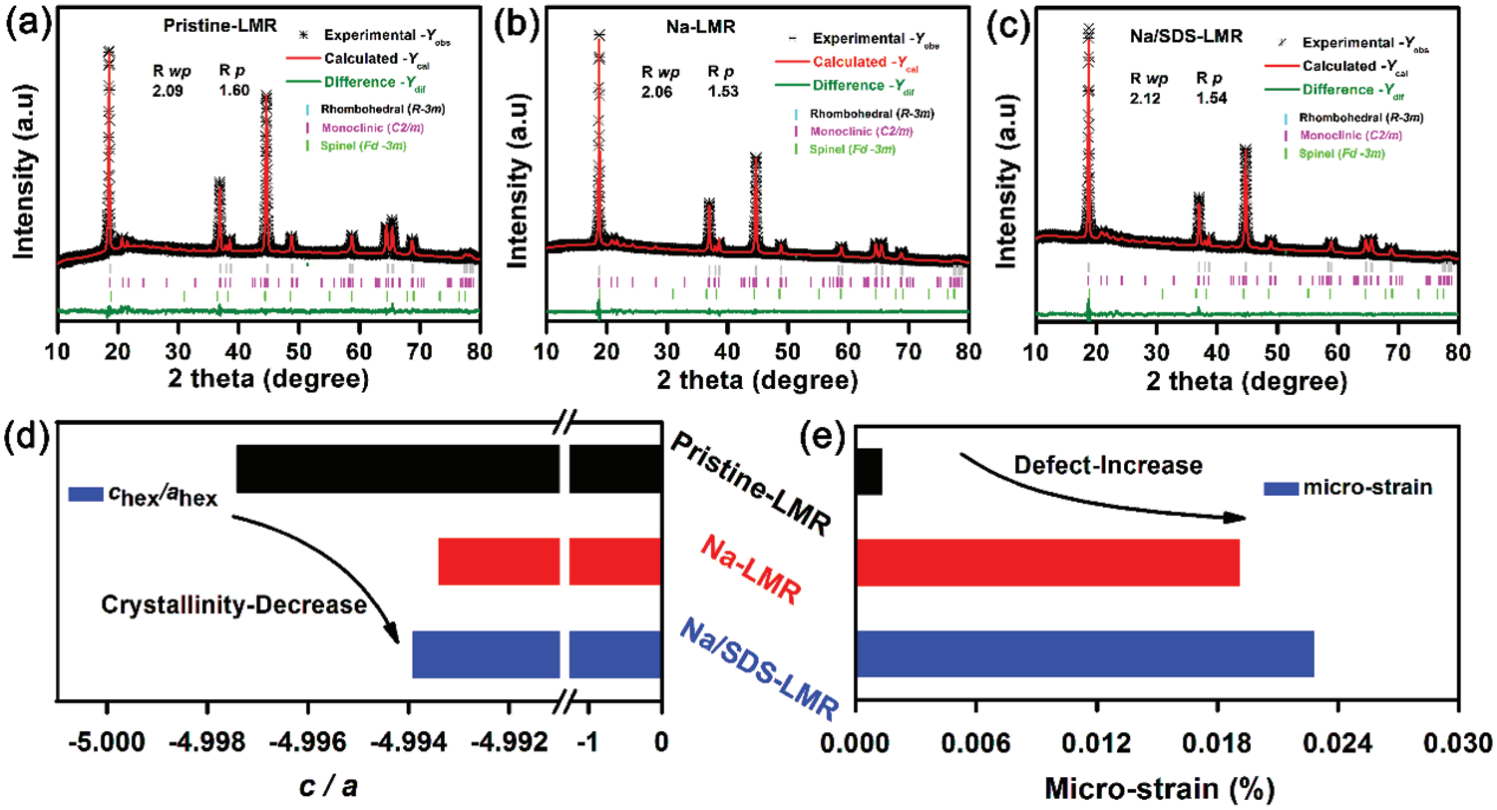
Powder XRD patterns with Rietveld refinement results: a) pristine‐LMR; b) Na‐LMR; c) Na/SDS‐LMR; d) the refinement was carried out using the rhombohedral *R‐3m* space group (cyan vertical tick marks), monoclinic *C2/m* space group (pink vertical tick marks), and spinel *Fd‐3m* space group (green vertical tick marks). The corresponding values of *c/a*; e) the micro‐strains were also vividly exhibited in forms of histograms.

There are several advantages of Na^+^‐doping that are listed below compared with pristine‐LMR. First, because of the larger ionic radius of Na^+^ in comparison to the lithium slabs, the inner doped Na^+^ would visibly increase the interplanar spacing of *c*‐axis, greatly weakening the diffusion resistance of lithium ions and charges, which is consistent with the results of XRD Rietveld refinement in **Figure** **1**. Second, the doped‐cations (Na^+^) would offer a certain amount of positively charged centers to stabilize the structure, hinder the TMs migration and inhibit the phase transition (layer to spinel and then rock‐salt structure) during the prolonged cycles.^16^ In Figure S4a, Supporting Information, the XRD patterns of NCMCO are indexed based on TMs carbonate and the XRD peaks of NCMO match well with NiMnO_3_. But there are some impurity peaks (marked as pink vertical lines), existing in Figure S4b, Supporting Information, which might be related to the coating layer that formed on the surface. By matching with the standard XRD patterns of possible impurities, these extra peaks are confirmed as Na_2−_ *_x_*Mn_8_O_16_ (0.4 < 2−*x* < 1), denoted as NMO hereafter. As shown in **Table** **2**, the (001) crystal planes of pristine‐LMR, Na‐LMR, and Na/SDS‐LMR with an increased refined interplanar distance benefit ion diffusion. Moreover, the increased micro‐strain (from 0.0013% for pristine‐LMR to 0.028% for Na/SDS‐LMR) as shown in Figure 1d and the minished value of c/a (from 4.9974 for Pristine‐LMR to 4.9939 for Na/SDS‐LMR, yet the closer the ratio is to 5, the better the layered structure is maintained) as shown in Figure 1e, indicating a growth of defects and a decrease of crystallinity.^41^ Hence, combining with the SEM images of the final products in Figure S5, Supporting Information, which shows a gradually recognizable grain boundaries from pristine‐LMR, Na‐LMR to Na/SDS‐LMR, the defective system may result from the short range lattice distortion caused by the slight inner micro‐strain and the outer structure that tends to be hierarchical distinctly.

**Table 2:** Detailed XRD Rietveld refinement results of pristine‐LMR, Na‐LMR, and Na/SDS‐LMR samples

Sample	*a*_hex_ [Å]	*c*_hex_ [Å]	*c*_hex_/*a*_hex_	Microstrain [*%*]
Prestine‐LMR	2.8444	14.2125	4.9974	0.0013
Na‐LMR	2.8506	14.2342	4.9934	0.0191
Na/SDS‐LMR	2.8478	14.2217	4.9939	0.0228

**Figure 2:**
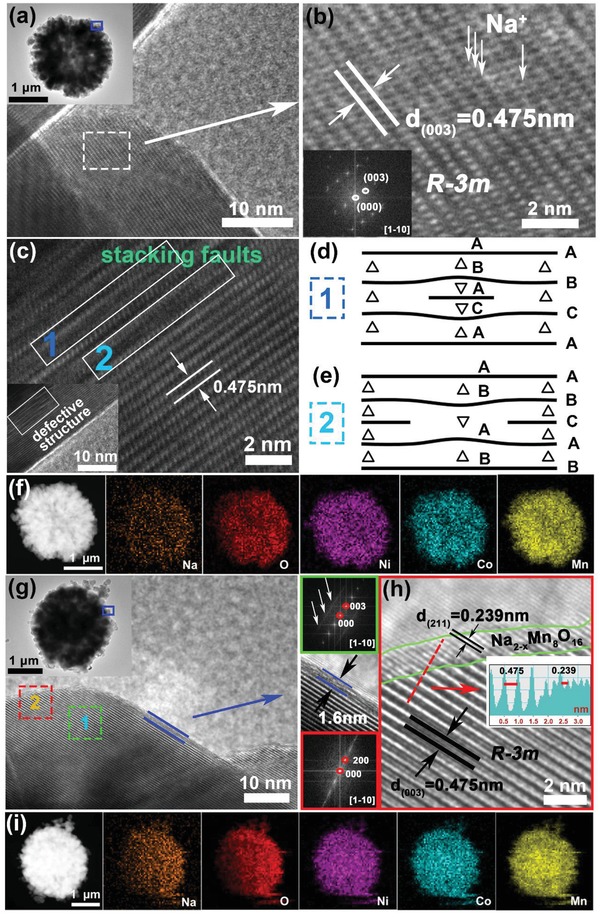
a) HRTEM image of Na/SDS‐LMR with the TEM image of secondary particle (inset); b) enlarged view of white square region and corresponding FFT pattern of Na/SDS‐LMR (inset); c) the HRTEM image of Na/SDS‐LMR with stacking faults in regions 1 and 2 which can be easily seen; d,e) two types of stacking faults—insertion layer like sequence AB A CAB and the deletion layer like sequence ABC AB ABC, respectively; f) the EDS element mappings of Na/SDS‐LMR; g) the HRTEM image of Na‐LMR with corresponding secondary particle (inset); h) the enlarged view of region 2 with the corresponding FFT patterns of region 1 (green square) and region 2 (red square); i) the EDS element mappings of Na‐LMR.

**Figure 3:**
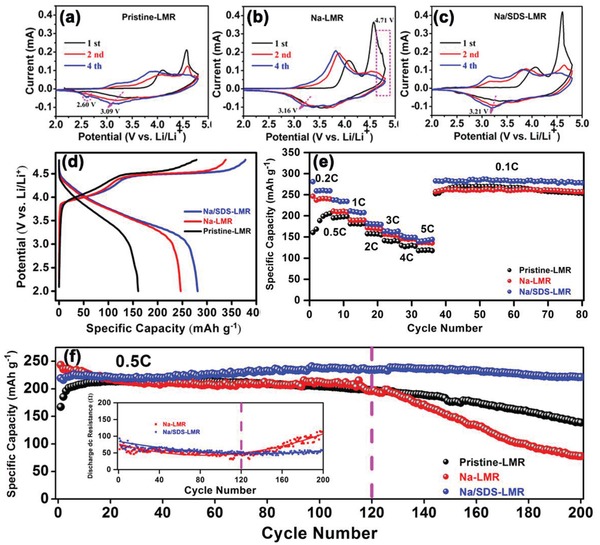
a–c) The CV curves of pristine‐LMR, Na‐LMR and Na/SDS‐LMR, respectively; d) charge‐discharge profiles for the first cycle of pristine‐, Na‐, and Na/SDS‐LMR in the voltage range 2.0–4.8 V at 0.1C rate (1C = 200 mA g^−1^) and room temperature; e) rate performance of pristine‐, Na‐, and Na/SDS‐LMR electrodes at various current rates of 0.2, 1, 2, 3, 4, 5, and 0.1C rates after the first activation cycle at the 0.1C rate; f) cycling stabilities of cathodes at 0.5C rate and the discharge dc inner resistance plots for Na‐LMR and Na/SDS‐LMR electrodes (inset at bottom left).

To further observe the detailed features of electrode materials and confirm the phase composition and the defects of cathodes, HRTEM and scanning transmission electron microscopy (STEM) investigations were carried out. The inset in the upper left corner of **Figure** **2**a is the HRTEM image of micro‐sized secondary particles for Na/SDS‐LMR. Figure 2b is the enlarged view and the corresponding fast Fourier transformation (FFT) pattern of blue square region which can refer to an extremely pure layer phase of *R‐3m* with a lattice spacing of 0.475 nm, corresponding to the typical (003) plane of layered α‐NaFeO_2_ structure. And it is clearly witnessed that the Na^+^ ions are deep‐doped into inner lattices (marked by the white arrows in Figure 2b) not forming a coating layer on the surface. A defective structure can be found in Figure 2c of the Na/SDS‐LMR, the stacking faults are labeled with rectangles.^42^ The two types stacking faults that insertion layer like sequence CAB A CAB and the deletion layer like sequence ABC AB ABC are vividly shown in **Figure** **3**d and Figure 3e, respectively;^43^ furthermore, the pictures in the upper left corner of Figure 2g are the HRTEM images of micro‐sized secondary particles for Na‐LMR. Figure 2h is the enlarged view of red square region 2. The FFT patterns of regions 1 and 2 demonstrate two sets of lattice fringes. Region 1 with a lattice spacing of 0.475 nm is consistent with (003) planes of pure layered α‐NaFeO_2_ structure (*R‐3m*).^44^ Besides, there is a distinguishable thin layer coated on the surface in region 2 with a uniform thickness of 1.6 nm and a different lattice spacing of 0.239 nm.^32^ Combined with our previous analysis of the XRD for Na‐NCMO (Figure S4b, Supporting Information), the coating layer is identified as NMO. In other words, the Na^+^ ions concentrate on the surface of Na‐LMR and formed NMOs, not all of them enter the interior of grains. The element mappings of Na/SDS‐LMR that are detected by energy dispersive spectrum (EDS) are illustrated in Figure 2f, vividly exhibiting a uniform distribution of Ni, Co, Mn, Na, and O. However, the distribution of Na and O elements for Na‐LMR (as shown in Figure 2i) is uneven, suggesting the aggregation effect of dopant, and the corresponding plots are shown in Figure S6, Supporting Information, and element contents are shown in Table S1, Supporting Information. Combining the phenomenon of surface coating and the segregation of sodium ions, it can be inferred that the distribution of coating layer may not be complete but partial. As a result, the protective effect may only affect the coated area not the inside bulk and other areas in direct contact with the electrolyte will become weak in resisting electrolyte corrosion.

To evaluate the improvement of electrochemical performance by defective structure, the electrode materials were tested at various current rates by assembling coin‐type half‐cells within the voltage range of 2.0–4.8 V. As shown in Figure 3a–c, the oxidation peaks at 3.8 V of cyclic voltammetry (CV) curves are corresponding to the slope region between 2.0 V and 4.45 V in Figure 3d, which represent the oxidation processes of Ni^2+^ to Ni^4+^, Co^3+^ to Co^4+^ and the deintercalation of Li^+^ from lithium‐layer to form octahedral vacancies. Furthermore, the plateau region at 4.5 V corresponds to the activation process of Li_2_MnO_3_ (Li_2_MnO_3_ → Li_2_O + MnO_2_) and the deintercalation process of Li^+^ from both lithium‐layers and metal‐layers, accompanied by the oxidation of O^2−^ (like O^2−^/O^−^ or O^2−^/O_2_) which corresponds to the strongest peak at about 4.6 V in CV curves.^34^ Obviously, the capacities of slope region are almost the same (about 160 mAh g^−1^), but the capacities of the plateau region increase with the doping of Na^+^ (137, 194, and 217 mAh g^−1^ for pristine‐LMR, Na‐LMR, and Na/SDS‐LMR, respectively), which may be caused by the charge compensation. These positively charged ions may lead to the generation of a few Mn^3+^ ions in systems to balance the excess charge of doped sodium ions, which is confirmed by the X‐ray photoelectron spectroscopy (XPS) analysis in Figure S7, Supporting Information.^45^, ^46^, ^47^ In addition, the rate of performance and cycling stabilities are shown in Figure 3e,f. Compared with the pristine‐LMR, the activation effect is alleviated in Na‐LMR and even disappears completely in Na/SDS‐LMR, which originates from the good contact of solid–liquid interface and the large specific surface area. This phenomenon is also due to the decreased amount of the formed Li_2_CO_3_ over the prolonged cycles which is verified in Figure S8, Supporting Information.^6^, ^44^, ^48^ As illustrated in Figure 3f, the prolonged cycles (120 cycles) of LMR oxide cathodes have been promoted dramatically by the uniform Na^+^‐doping and defective structures; the electrode of Na/SDS‐LMR maintain 93.1% of capacity (221.5 mAh g^−1^) at 0.5C rate after 200 cycles while only 64.8% (139 mAh g^−1^) is retained for the pristine‐LMR.

As shown in Figure S9, Supporting Information, the problem of voltage fading is also suppressed and agrees with the curves of dQ/dV (Figure S10, Supporting Information) and the reduction peaks marked by pink arrows in Figure 3a–c, especially after 120 cycles. The electrochemical impedance spectroscopy (EIS) measurements of cathodes before and after 50 cycles at 2C rate and the fitting results of Li^+^ ions diffusion coefficients (*D*_Li_) are shown in Figures S11 and S12, Supporting Information, respectively. Compared to pristine‐LMR, the cation uniformly doped electrode, Na/SDS‐LMR, gets a smaller increase of impedance after cycling. Also, the *D*_Li_s of electrodes after 50 cycles are summarized in Table S2, Supporting Information, and the Na/SDS‐LMR exhibits a better charge–ion transfer kinetics, which is significantly promoted from 1.556 × 10^−16^ to 3.391 × 10^−16^ cm^2^ s^−1^, originating from the increased interplanar spacing of *c*‐axis and the boosted diffusion of conducting ions.^49^ However, unlike the conventional tendency of a tardily capacity decay happened to pristine‐ and Na/SDS‐LMR, there is a distinct sharp capacity decrease in the prolonged cycle of Na‐LMR and always occur around 120 cycles; the same case is also found in the specific discharge energy density (Figure S13, Supporting Information). The discharge dc inner resistance plots for Na‐LMR and Na/SDS‐LMR electrodes (inset in the bottom left corner of Figure 3f) demonstrate that when the prolonged cycle reaches about 120 cycles, the discharge dc inner resistance of Na‐LMR electrode appears to be in an abrupt transition from decreasing to increasing; however, the same condition does not occur in the electrode of Na/SDS‐LMR. The facts described above indicate that some detrimental reactions must have happened during prolonged cycles of Na‐LMR, which causes the increase in internal resistance. Combined with the previous analysis, this phenomenon could be attributed to the crack and collapse of the doping element segregation gathered surface. Furthermore, the protective effect of the Na^+^ doped in Na‐LMR primary grain particles is not complete and lasting due to its uneven distribution.

## 3 Mechanisms of Enhanced Performance

The coin‐cells after 200 cycles at 0.5C rate were carefully dismantled to gain the cathode plates to further explore the effect of surface coating layer (NMO) on electrochemical performance of Na‐LMR electrode during prolonged cycles. The SEM images of pristine‐LMR, Na‐LMR, and Na/SDS‐LMR electrodes after 200 cycles are illustrated in Figure S14, Supporting Information. Compared with the electrodes before cycling in Figure S5, Supporting Information, pristine‐LMR after 200 cycles show a lot of dissolved and corroded primary particles, and the amorphous species that exist in the boundaries of primary grains is speculated as the products of side reactions like Li_2_CO_3_, LiF_3_ and so on.^37^ Na‐LMR after 200 cycles demonstrates numerous cracks, full of holes and execrably corroded pits, and the surface of secondary particles, unevenly and partially coated by NMO, may experience a severe rupture and cannot deliver protection during prolonged cycles, which might lead to phase transition. Na/SDS‐LMR electrode after 200 cycles shows intact primary particles and crevice‐free surface; the inner uniform‐doped Na^+^ ions effectively reinforce the electrode particle itself and suppress the corrosion effect of electrolyte.

**Figure 4:**
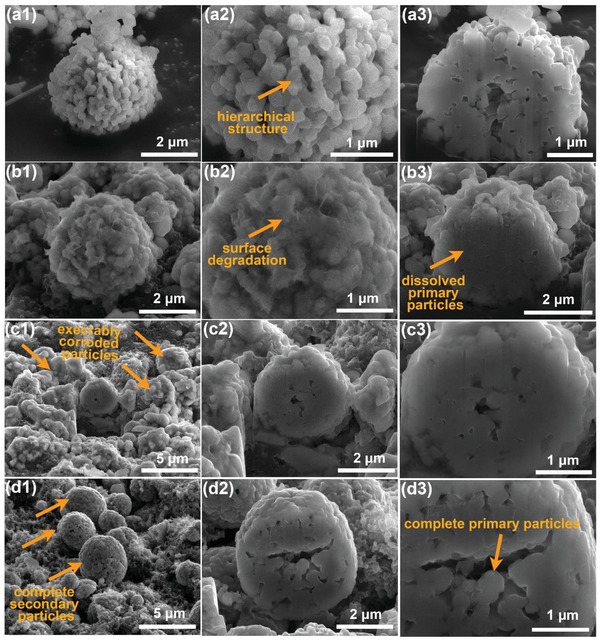
The cross‐sectional SEM images of a1–a3) for Na/SDS‐LMR secondary particles before cycling; b1–b3) for Pristine‐LMR secondary particles after 200 cycles at 0.5C rate; c1–c3) for Na‐LMR secondary particles after cycled and d1–d3) for Na/SDS‐LMR secondary particles after cycled.

The cross‐sectional SEM images of Na/SDS‐LMR secondary particle before cycling and pristine‐LMR, Na‐LMR, Na/SDS‐LMR secondary particles after 200 cycles are shown in **Figure** **4** to validate the protection effect from electrolyte corrosion. As shown in Figure 4a1–a3, the Na/SDS‐LMR secondary particle before cycling presents a hierarchical structure. Namely, a micron‐sized secondary particle is composed of plenty of nano‐sized primary particles. The SEM and cross‐sectional SEM images of pristine‐LMR in Figure 4b1–b3 show the dissolved primary‐particle cross section, wherein the surface morphology has been destroyed. However, the corresponding elements distribute evenly as revealed in Figure S15a1,a2, Supporting Information. The Na‐LMR secondary particle suffers a terrible surface corrosion during the prolonged cycles as illustrated in Figure 4c1–c3, which is consistent with the SEM images depicted in Figure S14, Supporting Information. In Figure 4d1–d3, the Na/SDS‐LMR secondary particles after 200 cycles still keep a complete secondary particles morphology without surface holes. As for the distribution of dopant of internal secondary particles, the element mapping of the cross section has been offered. The Na^+^ ions show uneven distribution and segregation in Na‐LMR electrode after prolonged cycles in Figure S15b1,b2, Supporting Information, which is consistent with Figure S16, Supporting Information. However, uniform distribution of Na^+^ ions in Na/SDS‐LMR secondary particles after 200 cycles can be seen clearly in Figure S15c1,c2, Supporting Information. Combining the above element distribution results and TEM results in Figure 2g,h, it can be concluded that the NMO coating layer on the surface of Na‐LMR is not a complete coating, but a partial and uneven coating. That is, the partially coated outermost layer (NMO) may act as a protective layer to avoid direct contact between electrolyte and electrode materials only at the coated area, and cannot protect the inside bulk and other weak areas that are still in direct contact with the electrolyte and thus suffer from electrolyte corrosion.

**Figure 5:**
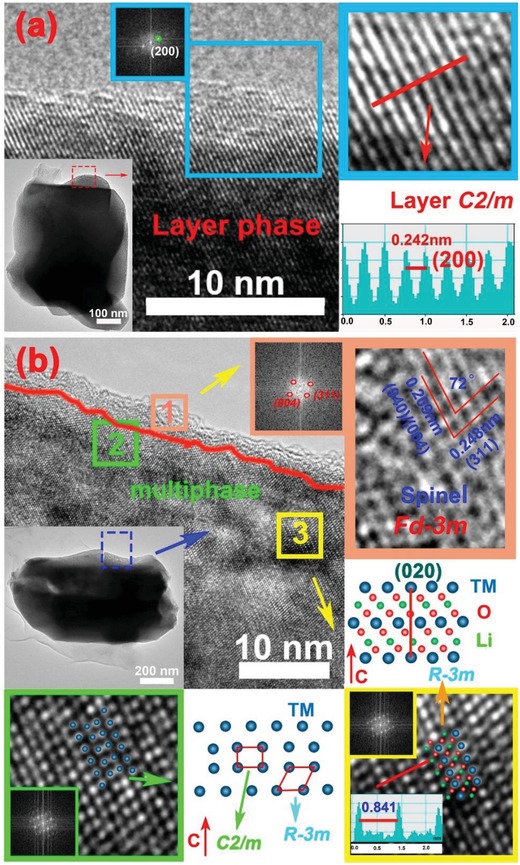
a) The HRTEM and STEM images of the primary particles and corresponding outermost surface morphology for electrodes of Na/SDS‐LMR after 200 cycles at 0.5C rate; b) electrodes of Na‐LMR after 200 cycles at 0.5C rate. The Na/SDS‐LMR electrode after 200 cycles still kept a pure layered phase while there were three sets of lattice fringes presented in the electrode of Na‐LMR after 200 cycles. Region 3 (yellow square) can be referred to layered α‐NaFeO_2_ structure (*R‐3m*), region 2 (green square) was identified as the coexistence of monoclinic Li_2_MnO_3_ phase (*C2/m*) and α‐NaFeO_2_ structure (*R‐3m*), and region 1 (orange square) consisted of the spinel phase *Fd‐3m*.

Additionally, the HRTEM images of electrodes after 200 cycles show distinctively different structural features in **Figure** **5**. The images of Na/SDS‐LMR electrode after 200 cycles still keep a pure layered phase as shown in Figure 5a. Meanwhile, the FFT pattern and the interplanar spacing of 0.242 nm are consistent with (200) plane of *C2/m*. What's more, the intact layered structure is still kept even on the outermost surface of primary grains, indicating an effective inhibition of phase transition. However, there are three sets of lattice fringes in the images of Na‐LMR after 200 cycles in Figure 5b. The interior lattice fringes with an interplanar spacing of 0.42 nm and the schematic structures of region 3 (yellow square) refer to the (020) plane of layered α‐NaFeO_2_ structure (*R‐3m*). However, region 2 (green square) indicates a hetero‐interface of the intergrowth two‐phase; both monoclinic Li_2_MnO_3_‐like phase (*C2/m*) and layered α‐NaFeO_2_ structure (*R‐3m*) are verified. The spinel phase *Fd‐3m* is observed on the outermost surface in region 1 (orange square). The angle of 72° and corresponding interplanar spacings are consistent with (004)/(040) and (311) planes of spinel structure, suggesting that the falling apart of the NMO layer on the surface results in a terrible phase transition during prolonged cycles, which is consistent with the analysis of results in Figure S17, Supporting Information. 50, 51

**Figure 6:**
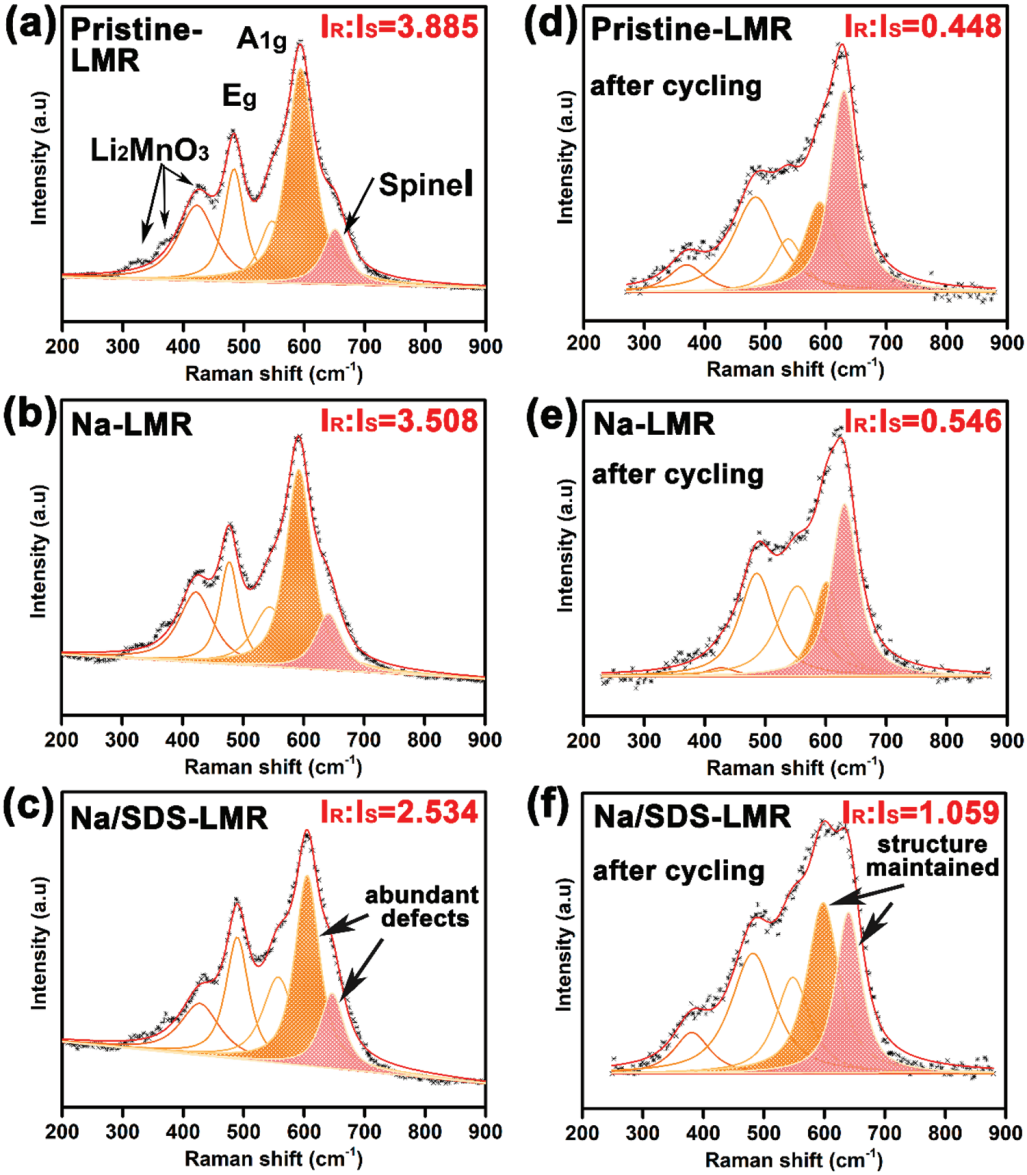
The Raman spectra and fitted results of pristine‐LMR, Na‐LMR, and Na/SDS‐LMR samples before and after cycling. a–c) The Raman spectra and fitted results of three types of electrodes before cycling. d–f) The Raman spectra and fitted results of three types of electrodes after 200 cycles at 0.5C rate.

As shown in **Figure** **6**, the Raman spectra and corresponding fitted results of pristine‐LMR, Na‐LMR, and Na/SDS‐LMR electrodes before and after cycling are employed to further explore the mechanism of the enhanced performance. Because of its excellent sensitivity of ordered structure, the Raman spectra are always applied to analyze micro‐zone phase structures.^52^ Obviously, there are three main peaks in the Raman spectra of pristine‐LMR, Na‐LMR, and Na/SDS‐LMR electrodes before cycling in Figure 6a–c. The first narrow peak at about 430 cm^−1^ along with other weak peaks at about 330 cm^−1^ and 375 cm^−1^ are recognized as the vibration of Li_2_MnO_3_ components. The remaining two peaks with high intensity at nearly 495 cm^−1^ and 609 cm^−1^ are signed to E_g_ (O—M—O bending) and A_1g_ (M—O stretching) vibrations of the layered structure with *R‐3m* space group, respectively.^29^, ^53^ The spinel‐like structure is identified by the peak at about 647 cm^−1^ in the fitted results of pristine‐LMR, Na‐LMR, and Na/SDS‐LMR.^54^ The intensity ratio of layered structure (*I*_R_, the golden yellow region) and spinel‐like structure (*I*_S_, the light‐red region) before cycling decreases from 3.885 and 3.058 to 2.534. As a matter of fact, the higher the ratio (*I*_R_/*I*_S_), the better the layered structure; the lower the ratio (*I*_R_/*I*_S_), the richer the defective structures.^55^ Therefore, the as‐prepared pristine‐LMR samples keep a slightly better layered structure while the as‐prepared Na/SDS‐LMR electrode contains lots of nano‐defects (like stacking faults which can provide a dramatically enhancement for electrochemical performance). Nevertheless, the fitted ratio (*I*_R_/*I*_S_) of pristine‐LMR, Na‐LMR, and Na/SDS‐LMR electrodes after 200 cycles at 0.5C rate (shown in Figure 6d–f) increases correspondingly from 0.448 and 0.546 to 1.059; the highest (*I*_R_/*I*_S_) ratio of layer/spinel in Na/SDS‐LMR electrode after 200 cycles indicates an excellent structure stability and effective suppression of phase transition by SDS‐assisted uniform Na^+^‐doping method. On the contrary, a deal of well‐defined layered structures in pristine‐LMR and Na‐LMR are converted into spinel phase after prolonged cycles.

**Figure 7:**
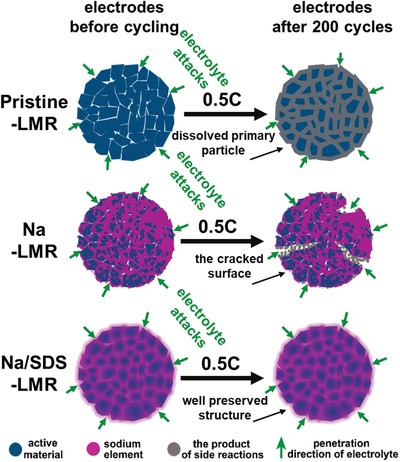
The schematic illustration for the evolution of the electrode structures and the inner penetration of electrolyte on the cross section of secondary particles.

It is feasible to preserve the integrity of secondary electrode particles by SDS‐assisted uniform doping method. As schematically illustrated in **Figure** **7**, the pristine‐LMR without any protective treatment would not resist the erosion of electrolyte; the formation of side reaction products between liquid electrolyte and solid cathodes interface during prolonged cycles would induce the TM^n+^ (Ni, Co, Mn) dissolution and structural degradation. As for Na‐LMR, the partially coated outermost layer (NMO) may act as a protection layer to avoid direct contact between electrolyte and electrode materials. However, this protection does not last too long, and it hardly protects the inside bulk and other areas that are still in direct contact with the electrolyte. Usually, the NMO layer would fall apart and disappear after 120 cycles, accompanied by sharp decreased capacity and sudden increased impedance. The Na/SDS‐LMR prepared by SDS‐assisted coprecipitation process guarantees a uniform distribution of Na^+^ ions in the inner region of electrode particles and also induces a wealth of defects. The possible fundamentals of improving performance by defective structure are mainly reflected in two places: first, the induced defects acting as a nail to peg down the ordered phase, delivering an obstacle to the phase transforming from order to disorder, which can be interpreted as a “pinning effect”; second, the diffusion of Li^+^ are active and more facile in defective structures, promoting the performance, which can be explained as an “activating effect”.^55^, ^56^, ^57^ Overall, the effectively reinforced structure and the induced abundant‐defects boost a fast diffusion of lithium ions, enable a decent performance, and prevent the detrimental side reactions on the cathode–electrolyte interface to maintain a splendid integrity of secondary particles.

## 4 Conclusion

In general, we present a workable strategy to inhibit the detrimental corrosion effect that occurs at the solid–liquid interface by preparing a uniform Na^+^‐doped and defective LMR. Consequently, the segregation and aggregation phenomena of Na^+^ ions are alleviated with the addition of the surfactant. Na^+^ ions are distributed in the intracell sites of cathode grains and they induce the formation of abundant stacking faults not just gathering on the surface, which may result from the weakened strength of Na—TM—O bonds by the amphipathicity of SDS. Moreover, the cooperation of cationic doping and defective structure provides an effective enhancement for the structure stability and electrochemical performance. As a result, a high reversible discharge specific capacity of 221.5 mAh g^−1^ after 200 cycles at 0.5C rate with a 93.1% of capacity retention for Na/SDS‐LMR are obtained, which is much better than that of pristine‐LMR (64.8%). The uniform Na^+^‐doping and defective structure can be extended to other doping systems and other types of surfactants, such as using sodium dodecylbenzene sulfonate (SDBS, which has similar properties to SDS) for Na^+^ doping and magnesium alkylbenzene sulfonate for Mg^2+^ doping and so on, which may provide a meaningful reference for the subsequent improvement of advanced battery materials.

## 5 Experimental Section

*Materials Synthesis*: To reveal the effects of SDS and Na‐doping, three experiments were set up with the experimental conditions of undoped, Na‐doped, and SDS‐assisted Na‐doped, respectively. Manganese chloride tetrahydrate (MnCl_2_·4H_2_O), nickel chloride hexahydrate (NiCl_2_·6H_2_O), cobalt chloride hexahydrate (CoCl_2_·6H_2_O), sodium carbonate anhydrous (Na_2_CO_3_), ammonium carbonate ((NH_4_)_2_CO_3_), and anhydrous lithium carbonate (Li_2_CO_3_) were purchased from Xilong Scientific Co., Ltd. (Shantou, China). Sodium dodecyl sulfate (SDS, AR) was purchased from Macklin Biochemical Co., Ltd. (Shanghai, China). All chemicals are analytically pure and without further treatment. The final Li‐ and Mn‐rich cathode materials Li_1.2_Ni_0.13_Co_0.13_Mn_0.54_O_2_ (LMR) were prepared by a typical co‐precipitation method and the subsequent high temperature calcination process. Take the experiment three, for example. First, 1.8 g SDS powder was added into the deionized water and was stirred for 5 min. At the same time, the stoichiometric amounts of TM (Ni, Co, Mn) chlorate were dissolved into deionized water to constitute a solution with a concentration of 0.08 m and subsequently dropwise added into the SDS solution. Then, a 0.2 m Na_2_CO_3_ solution was added into the previous mixed solution slowly and kept stirring for 12 h in a water bath at 26 °C throughout the whole process. Subsequently, the precipitate was collected by centrifugation from suspension solution, and then washed with deionized water and absolute ethyl alcohol three to five times. Afterward, the SDS‐assisted Na‐doped carbonate precursors Ni_0.13_Co_0.13_Mn_0.54_(CO_3_)_0.8_ (Na/SDS‐NCMCO) were obtained after being dried overnight in a drying box kept at a constant temperature of 80 °C. Second, the Na/SDS‐NCMCO was preheated at 500 °C in air (heating rate is 1 °C min^−1^) for 6 h to obtain oxide precursors Ni_0.13_Co_0.13_Mn_0.54_O_0.8_ (denoted as Na/SDS‐NCMO). Finally, a certain weight of anhydrous Li_2_CO_3_ (excess 3%) was mixed with NCMO and further annealed at 800 °C (heating rate is 2 °C min^−1^) for 12 h to receive the Na/SDS‐LMR product.

The first experiment was in a blank control group; in order to eliminate the influence of sodium ions, ammonium carbonate was used as precipitator. The carbonate precursor, oxide precursor, and the final product were denoted as pristine‐NCMCO, pristine‐NCMO, and pristine‐LMR, respectively. In experiment two, only the precipitator was changed into sodium carbonate. The corresponding carbonate precursor, oxide precursor, and the final product were denoted as Na‐NCMCO, Na‐NCMO, and Na‐LMR, respectively.

*Structure Characterizations*: The crystalline phase of the prepared electrode materials were examined by powder X‐Ray diffraction (XRD, Ultima IV‐185, Rigaku, Japan) using Cu Kα‐radiation (λ = 1.5406 Å) at a scan rate of 1° min^−1^ in the theta range of 10–100°, 25 mA and 40 kV. The lattice parameters were carried out by Rietveld refinement analysis, using the EXPGUI‐GSAS program.^51^, ^52^ The thermal gravimetric analyzer (TGA, SDT‐Q600) and Fourier transform infrared spectroscopy (FTIR, Nicolet‐is10) were applied to confirm the existence of SDS and carbonate. The Raman spectrum (Xplora) of electrode before and after long cycle was used to identify the phase change. The morphology and microstructure of the cathodes were characterized by field‐emission scanning electron microscopy (FESEM, SUPRA‐55, ZEISS, Germany). High‐resolution Tecnai F30 field emission gun transmission electron microscope (HRTEM, TECNAI‐F30, Philips‐FEI, Netherlands) was used to reveal the arrangement of atomic lattice by high‐resolution images and distribution of elements by mapping at a working voltage of 300 kV.

*Electrochemical Measurements*: The electrochemical tests with CR2032 coin‐type half‐cells were assembled in an Ar‐filled M Braun glove box (MB‐10‐G‐V2A, H_2_O and O_2_ < 0.5 ppm). The slurry that contained 80% of the active material, 10% polyvinylidene fluoride binder (PVDF), and 10% Super‐P was coated on an Al foil collector uniformly to prepare the cathode electrodes with an approximately 100 µm thickness and 1.8–2.5 mg cm^−2^ loading. The lithium metal foil was assembled as the anode electrode for the electrochemical half cells and 1 m LiPF_6_/ EC+DEC (1: 1 /v/v) as the electrolyte, and the Celgard 2500 polypropylene as separator. Cyclic voltammetry (CV) curves were tested by CHI660D electrochemistry workstation (Shanghai Chenhua Device Company, China) with a scan rate of 0.1 mV s^−1^; the electrochemical impedance spectroscopy (EIS) was measured with a frequency range of 100 kHz to 10 mHz. Charge–discharge and rate performance was performed between 2.0 and 4.8 V on the NEWARE battery testing systems (Shenzhen,China) after the first initial charge–discharge cycle at a 0.1C rate (1C = 200 mA g^−1^) at room temperature. In this work, the half cells after 200 cycles at 0.5C rate were carefully dismantled by hydraulic crimping machine (MSK‐110, Shenzhen, China) in Ar‐filled M Braun glove box to gain the cathode electrodes. The disassembled positive electrode plate was immersed in acetone immediately to dissolve the excess electrolyte and then transferred into anhydrous ethanol to wash out the organic solvent. The cleaned electrode plates were dried in 80 °C oven for subsequently various tests.

## Conflict of Interest

The authors declare no conflict of interest.

## Supporting information

Supplementary
