# Supplementary material for: Uniform Na+ Doping‐Induced Defects in Li‐ and Mn‐Rich Cathodes for High‐Performance Lithium‐Ion Batteries
Source: Adv Sci (Weinh). 2019 May 17;6(14):1802114. doi: 10.1002/advs.201802114 (PMC6661944; doi:10.1002/advs.201802114)
Supplement: Supplementary file 1 — Supplementary [file ADVS-6-1802114-s001.pdf]

Copyright WILEY-VCH Verlag GmbH & Co. KGaA, 69469 Weinheim, Germany, 2019.

ADVANCED SCIENCE Open Access

Supporting Information

for *Adv. Sci.,* DOI: 10.1002/advs.201802114

Uniform Na^{+} Doping-Induced Defects in Li- and Mn-Rich Cathodes for High-Performance Lithium-Ion Batteries

Wei He, Pengfei Liu, Baihua Qu, Zhiming Zheng, Hongfei Zheng, Pan Deng, Pei Li, Shengyang Li, Hui Huang, Laisen Wang, Qingshui Xie,* and Dong-Liang Peng**

Supporting Information

Title: Uniform Na^+ Doping-Induced Defects in Li- and Mn-rich Cathodes for High-Performance Lithium-ion Batteries

Wei He, Pengfei Liu, Baihua Qu, Zhiming Zheng, Hongfei Zheng, Pan Deng, Pei Li, Shengyang Li, Hui Huang, Laisen Wang, Qingshui Xie*, Dong-Liang Peng**

W. He, P. Liu, Dr. B. Qu, Z. Zheng, P. Deng, P. Li, S. Li, H. Huang, Prof. L. Wang, Prof. Q. Xie, Prof. D.-L. Peng

Department of Materials Science and Engineering, Collaborative Innovation Center of Chemistry for Energy Materials, College of Materials, and Pen-Tung Sah Institute of Micro-Nano Science and Technology, Xiamen University, Xiamen 361005, PR China

*Corresponding Author: *E-mail:bhqu@xmu.edu.cn;xieqsh@xmu.edu.cn; and dlpeng@xmu.edu.cn*

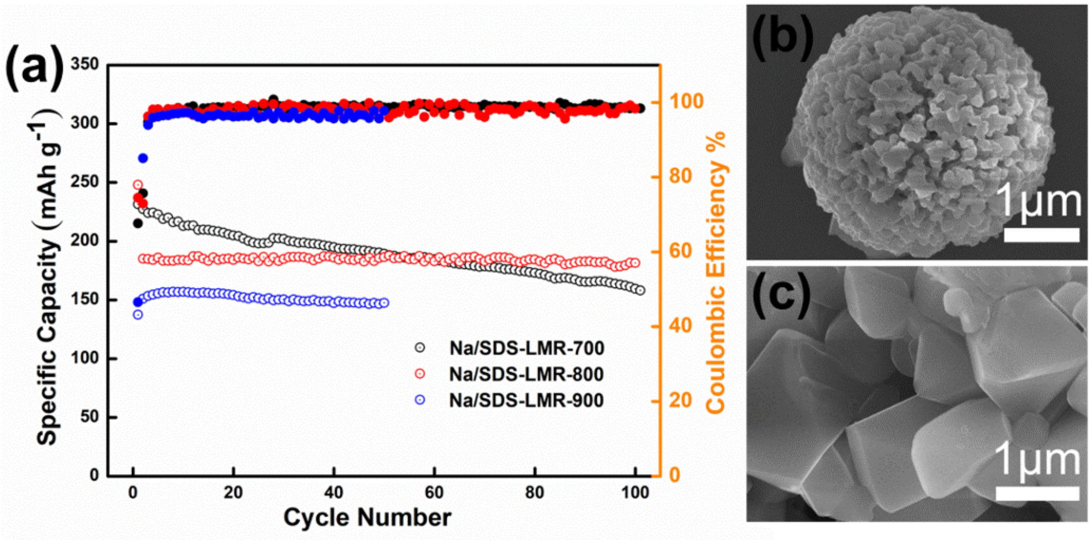

Figure S1. Effect of calcination temperature on the electrochemical performances and micro-morphology of Na/SDS-LMR. (a) cycling performance of Na/SDS-LMR as cathode prepared at different temperatures in the voltage of 2.0~4.8 V and at 2C rate (1C = 200 mA g^{-1}) at room temperature. (b), (c) SEM images of Na/SDS-LMR calcinated at 700°C and 900°C, respectively.

The FTIR curves of Pristine-NCMCO and Na-NCMCO were almost identical, excluding the effect of sodium ions (Fig. S2b). As for Na/SDS-NCMCO, the

absorption peaks at the wave number of 593 cm^{-1}, 995 cm^{-1}, 1250 cm^{-1} and 2982 cm^{-1} (indicated by green arrows in Fig. S2b) matched well with the standard infrared spectrum of pure SDS. These characteristic peaks of SDS could clearly notarize that the added-SDS was not washed away by deionized water and anhydrous ethanol, but still existed in the carbonate precursor Na/SDS-NCMCO.

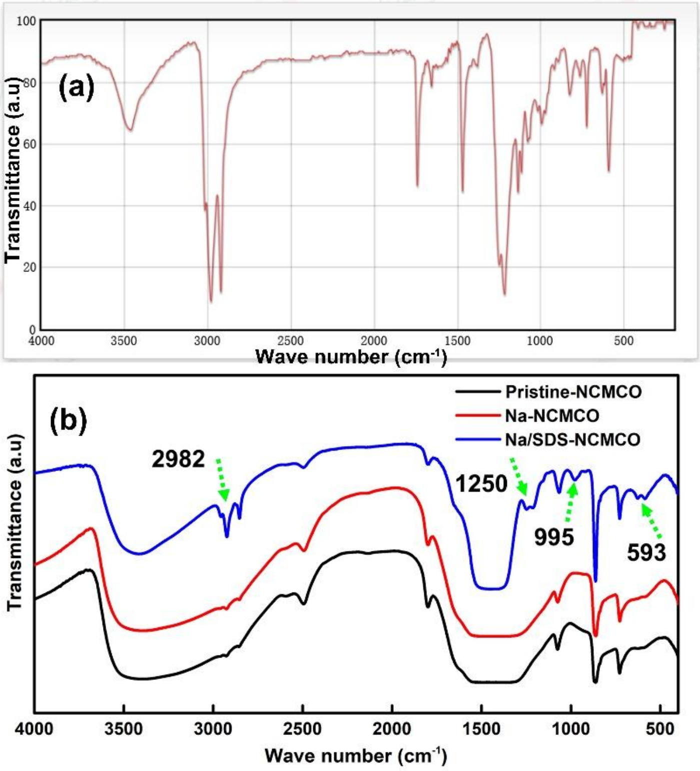

Figure S2. FTIR patterns of (a) pure SDS powders and (b) carbonate precursors of Pristine-NCMCO, Na-NCMCO, Na/SDS-NCMCO samples. The peaks indicated by green arrows in (b). were identified as the characteristic peaks of pure SDS.

The standard infrared spectrum of SDS in Fig (a). is quoted from: Shanghai Institute of Organic Chemistry of CAS. Chemistry Database [DB/OL]. http://www.organchem.csdb.cn.[1978-2018].

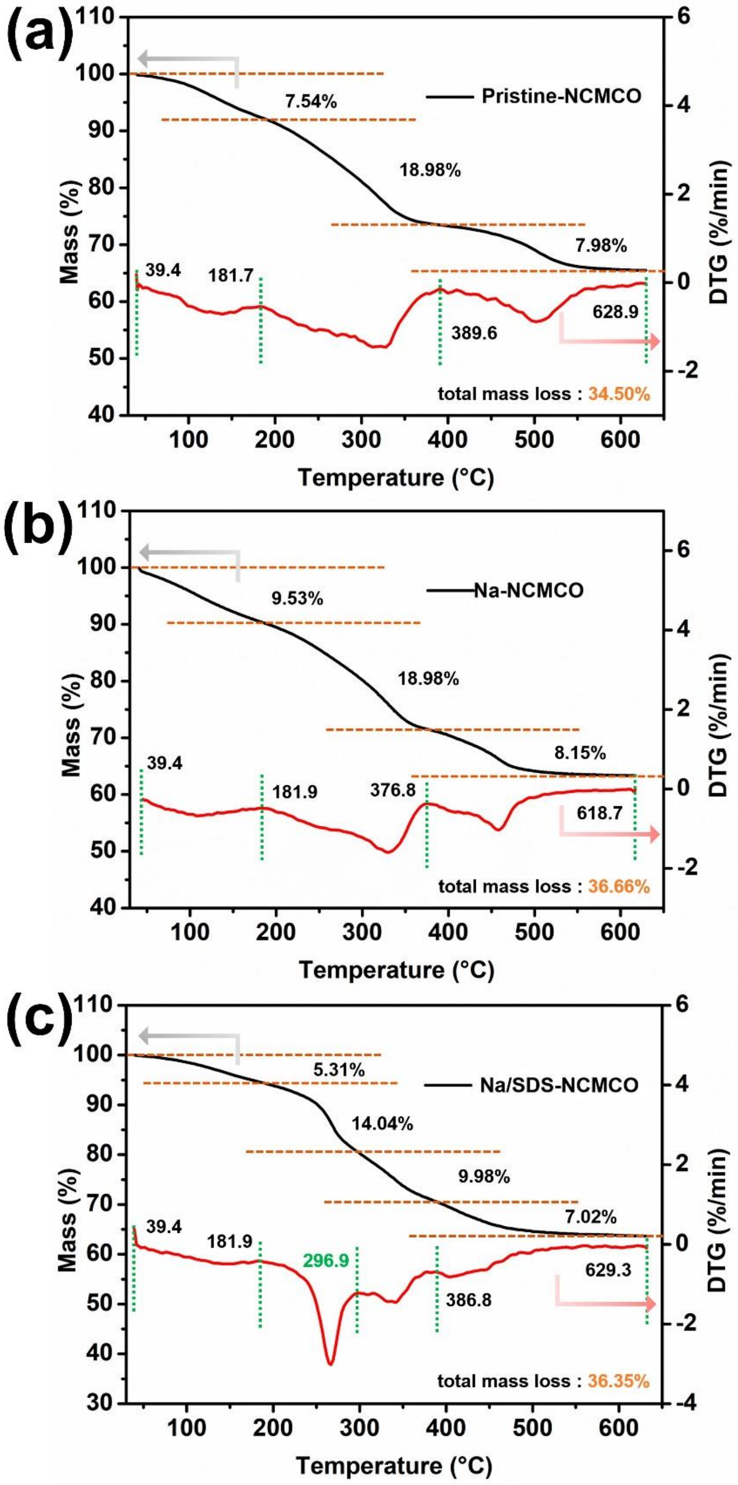

Figure S3. TG curves of the precursor Pristine-NCMCO (a), Na-NCMCO (b) and Na/SDS-NCMCO (c). The different stages of weight loss and the corresponding transition temperatures on the TG curves were separated by dashed lines.

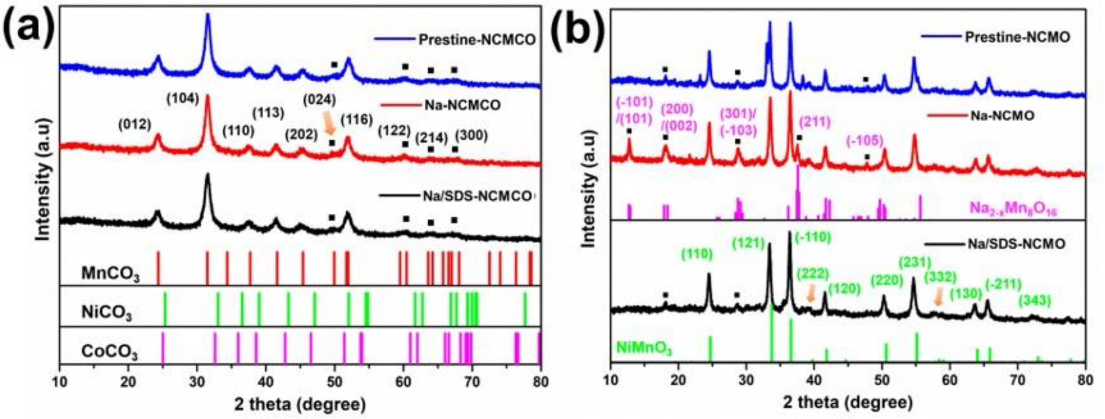

Figure S4. (a) XRD patterns of Pristine-NCMCO, Na-NCMCO and Na/SDS-NCMCO could be indexed based on carbonate of TMs ($NiCO_3$ PDF#12-0771, $MnCO_3$ PDF#44-1472, $CoCO_3$ PDF#11-0692). (b) XRD patterns of Pristine-NCMO, Na-NCMO and Na/SDS-NCMO matched well with $NiMnO_3$ (PDF#48-1330). Meanwhile, other peaks (marked as pink vertical-line) in Na-NCMO sample could be indexed into $Na_{2-x}Mn_8O_{16}$ (PDF#42-1347) in (b).

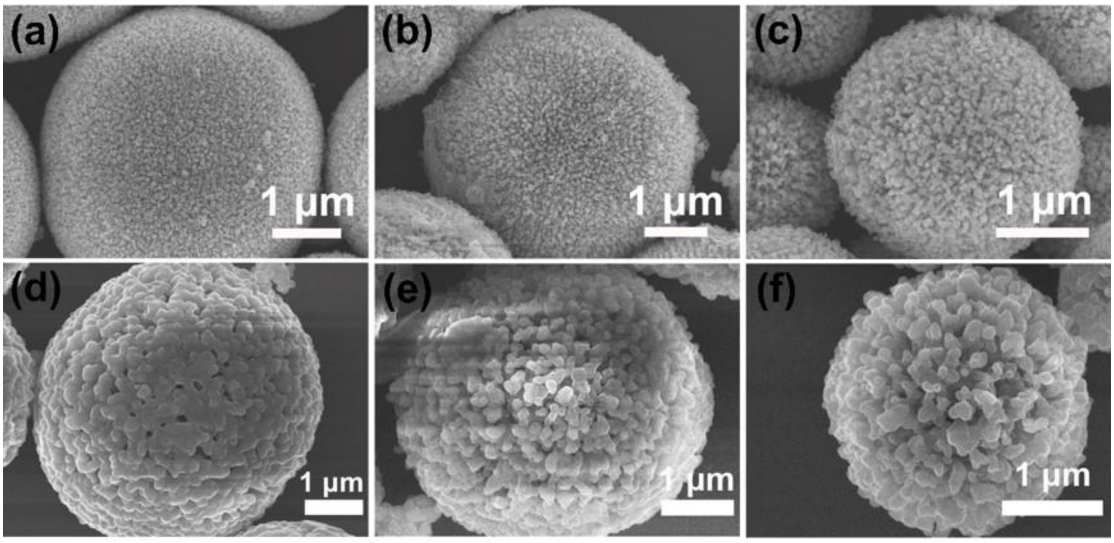

Figure S5. SEM images of the oxide precursors, (a) Pristine-NCMO, (b) Na-NCMO, (c) Na/SDS-NCMO. And SEM images of the final products before cycling (d) Pristine-LMR, (e) Na-LMR, (f) Na/SDS-LMR.

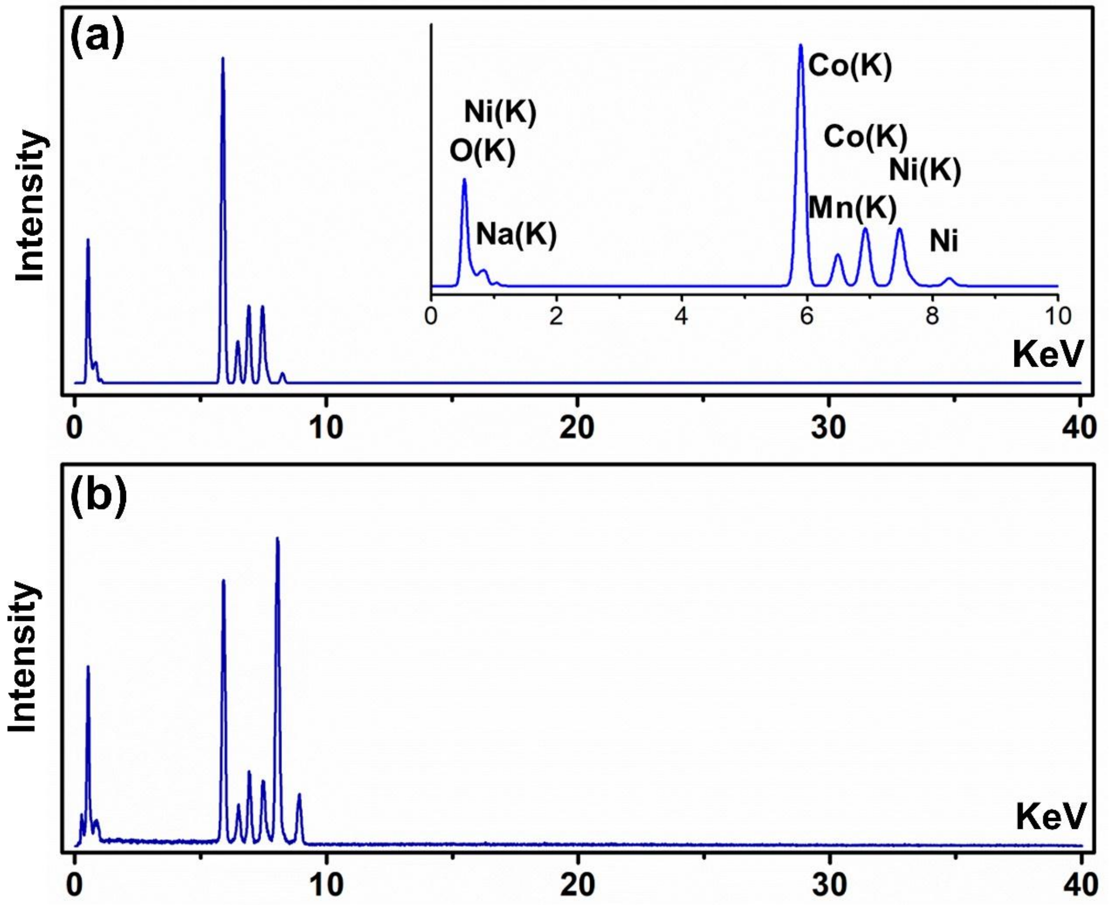

Figure S6. Energy Dispersive Spectrum plots of (a) for Na/SDS-LMR and (b) for Na-LMR.

Table S1. Summary of element content in Na/SDS-LMR and Na-LMR samples.

Sample / Element	Na/SDS-LMR Atomic percentage (%)	Na-LMR Atomic percentage (%)
**O(K)**	**46.615**	**58.127**
**Na(K)**	**0.589**	**0.94**
**Mn(K)**	**34.818**	**26.788**
**Co(K)**	**8.863**	**7.064**
**Ni(K)**	**9.112**	**7.078**
**Total (%)**	**100**	**100**

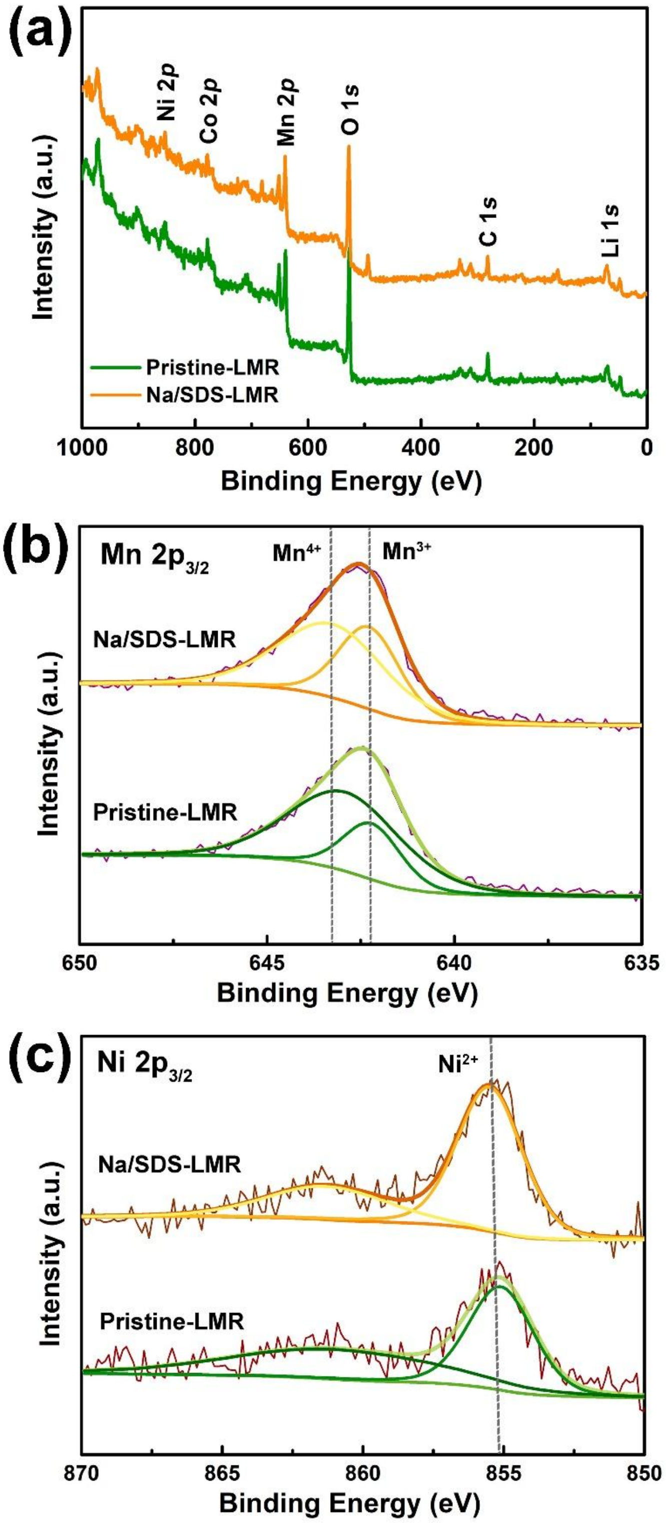

Figure S7. XPS spectra of Na/SDS-LMR and Pristine-LMR cathode materials, (a) the full-spectrum data, the XPS data of (b) Mn $2p_{3/2}$ and (c) Ni $2p_{3/2}$ indicating that the valance of Mn decreased due to balance the excess charge of doped cations while Ni kept nearly the same state.

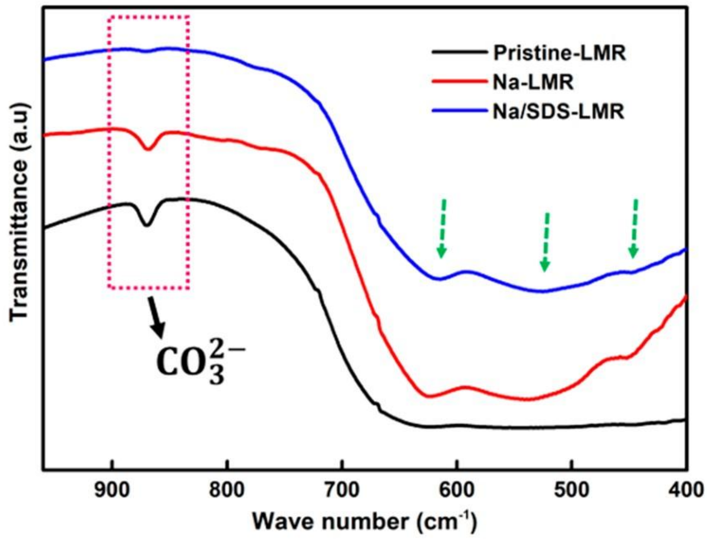

Figure S8. FTIR patterns of Pristine-LMR, Na-LMR and Na/SDS-LMR samples.

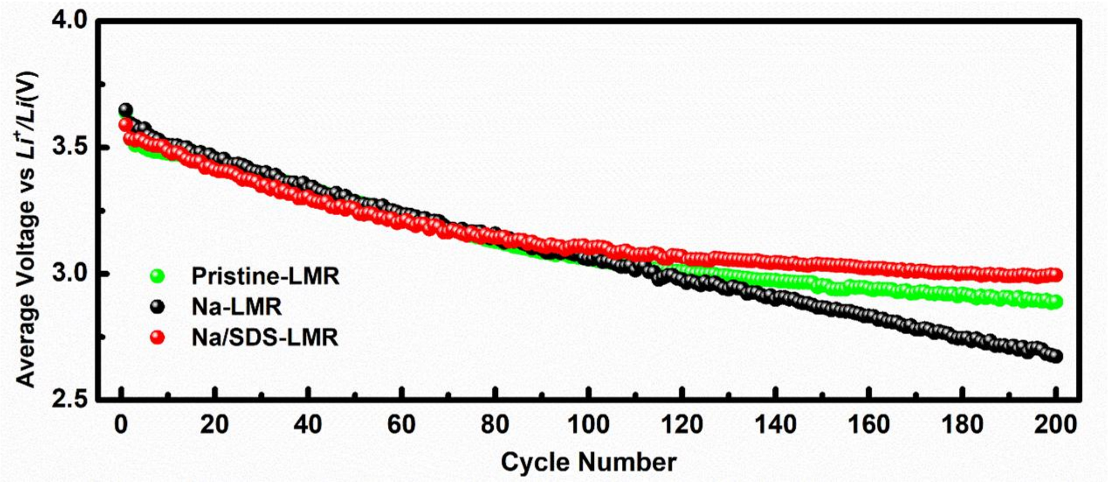

Figure S9. The curves of average discharge voltage during long cycle at 0.5C for Pristine-LMR, Na-LMR and Na/SDS-LMR electrodes.

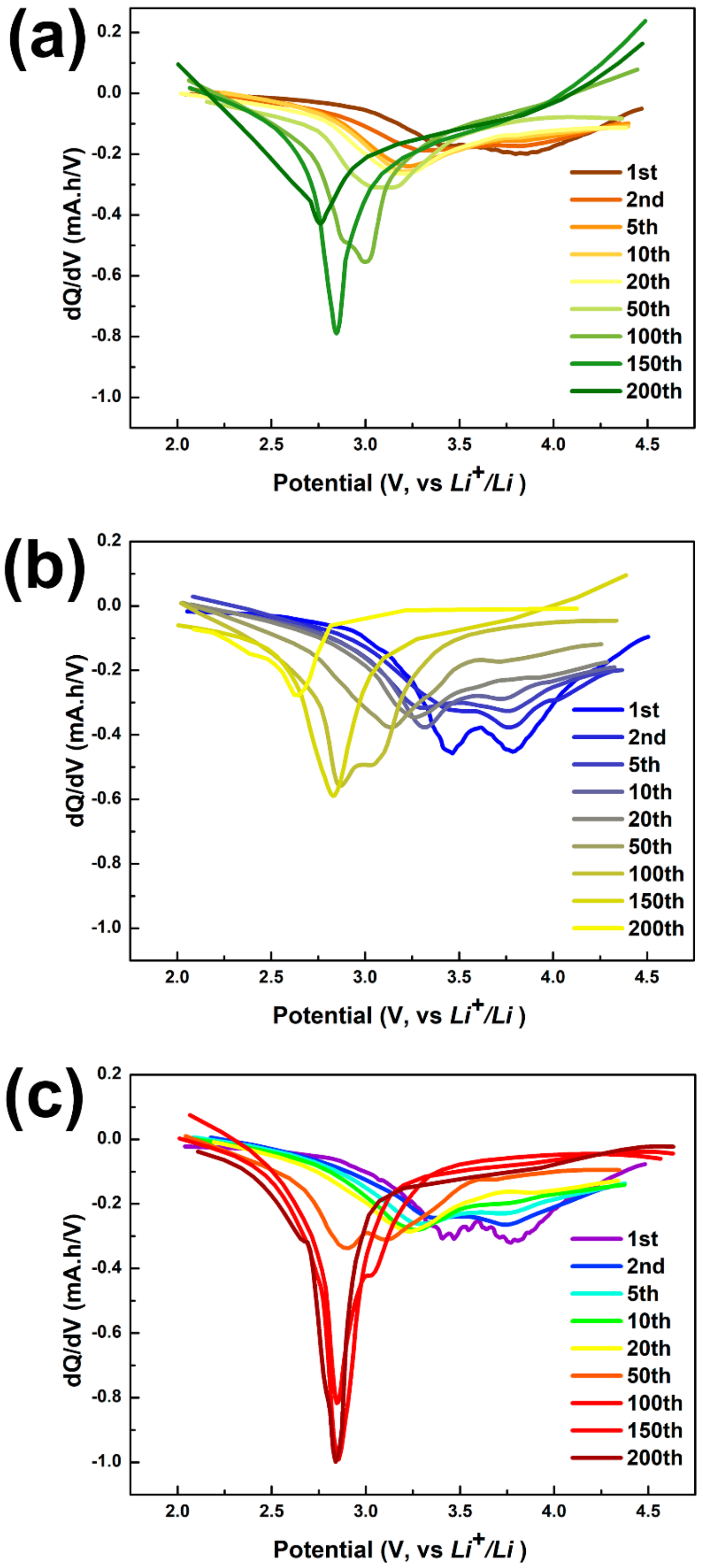

Figure S10. The curves of differential capacity versus voltage (dQ/dV) for Pristine-LMR, Na-LMR and Na/SDS-LMR cathodes at 0.5C.

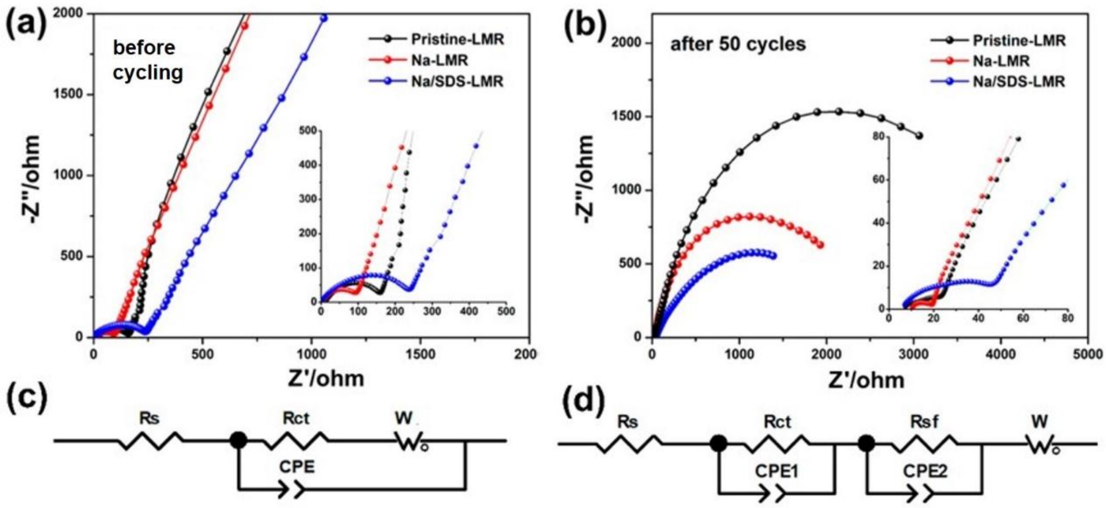

Figure S11. (a), (b), Nyquist Plots of Pristine-LMR, Na-LMR and Na/SDS-LMR cathodes before and after 50 cycles at 2C. The insets in (a), (b) are enlarged from the high-frequency region of the Nyquist Plots. (c) is the equivalent circuit used for fitting the impedance spectra before cycling and (d) is the equivalent circuit used for fitting the impedance spectra of after 50 cycles under 2C rate.

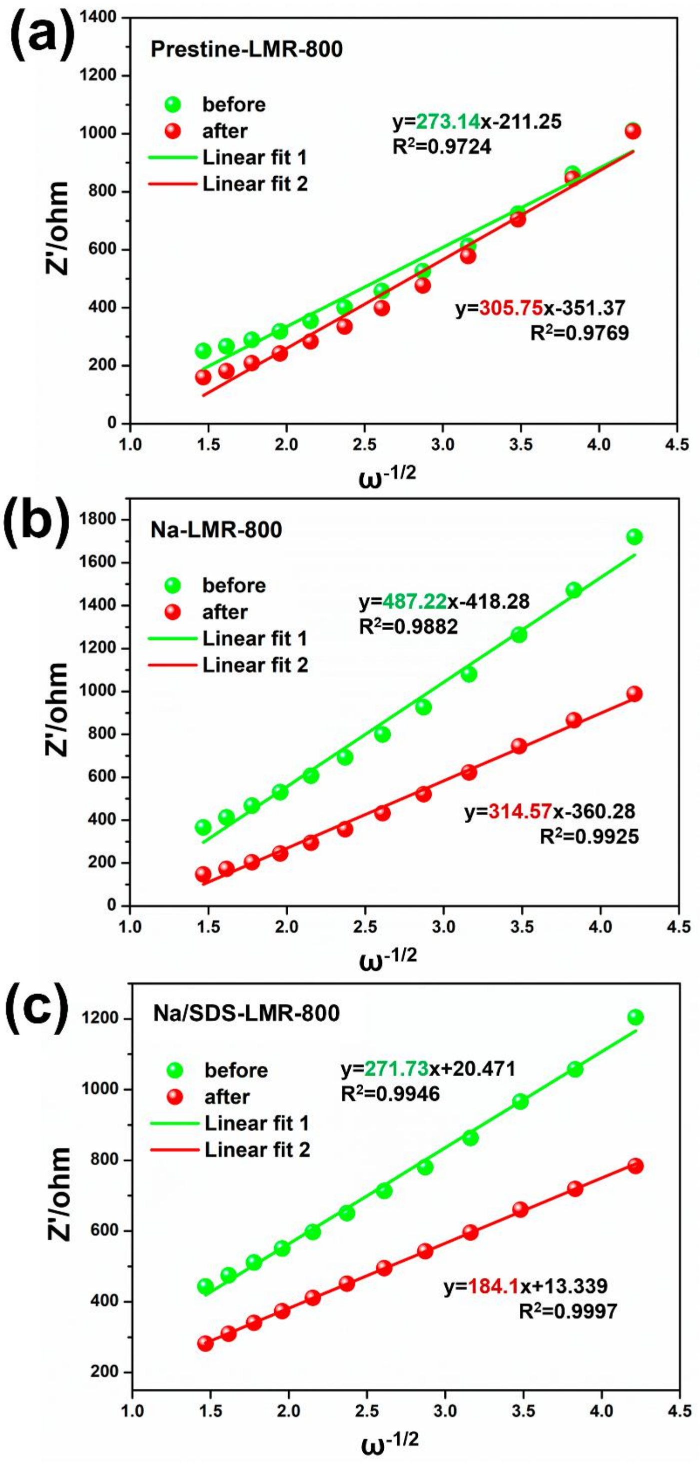

Figure S12. (a), (b), (c), relationships between Z' and $\omega^{-1/2}$ for Pristine-LMR, Na-LMR and Na/SDS-LMR samples before and after 50 cycles at 2C, respectively.

Table S2. The summary of lithium-ion diffusion coefficients (D_{Li}) before and after 50 cycles at 2C rate. (the unit of D_{Li} is $cm^2\ s^{-1}$)

Dopant	Additive	Sample	Before cycle	After 50 cycles	Variation trend
**No-doping**	**No-SDS**	**Pristine-LMR**	1.540×10^{-16}	1.229×10^{-16}	↓
**Na$^+$-doping**	**No-SDS**	**Na-LMR**	0.484×10^{-16}	1.161×10^{-16}	↑
	**With-SDS**	**Na/SDS-LMR**	1.556×10^{-16}	3.391×10^{-16}	↑

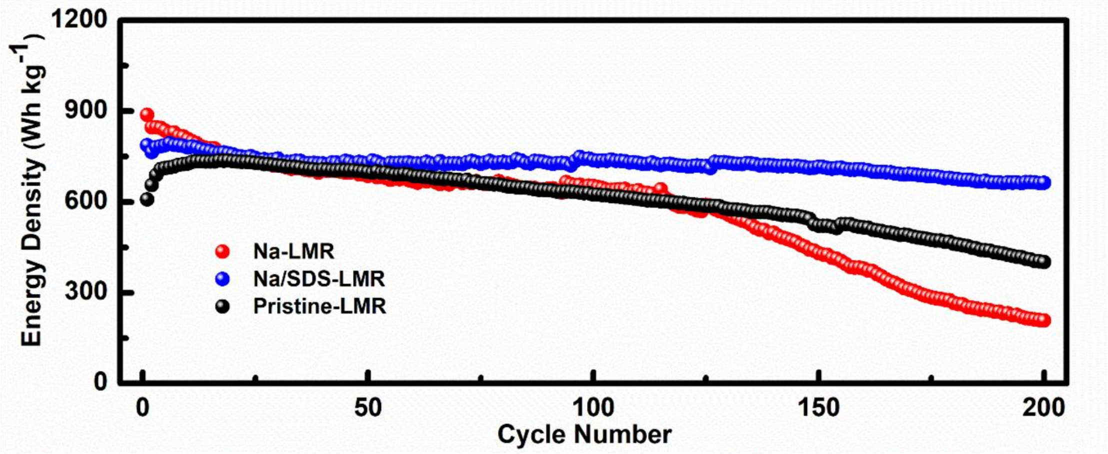

Figure S13. The curves of specific discharge energy densities during long cycles at 0.5C for Pristine-LMR, Na-LMR and Na/SDS-LMR cathodes.

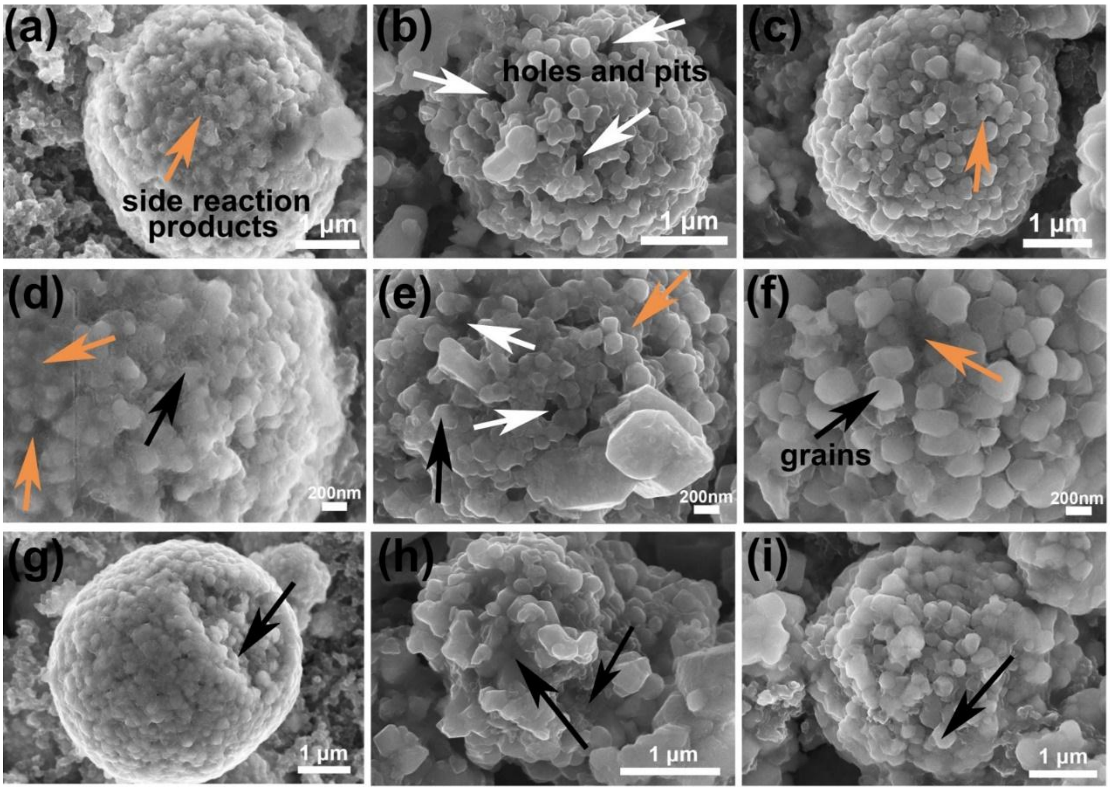

Figure S14. Morphology and surface SEM images of the cathode materials after 200 cycles at 0.5C rate. (a), (d), (g) Pristine-LMR electrode after 200 cycles shows dissolved primary particles and corroded pits (illustrated by black arrows), side reaction products (illustrated by orange arrows); (b), (e), (h) Na-LMR electrode shows lots of cracks, holes (illustrated by white arrows) and execrably corroded pits (illustrated by black arrows); (c), (f), (i) Na/SDS-LMR electrode after 200 cycles shows complete primary particles and surface without holes.

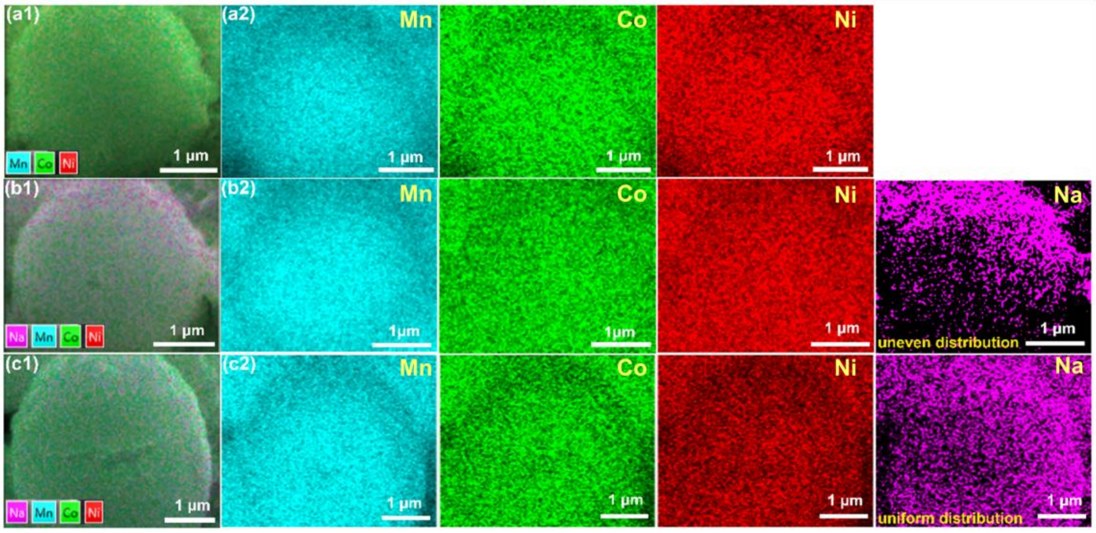

Figure S15. The cross-sectional SEM images of electrode secondary particles after 200 cycles and (a1) a2) the corresponding element mapping of the cross-section for Pristine-LMR, (b1) b2) the corresponding element mapping of the cross-section for Na-LMR, (c1) c2) the corresponding element mapping of the cross-section for Na/SDS-LMR.

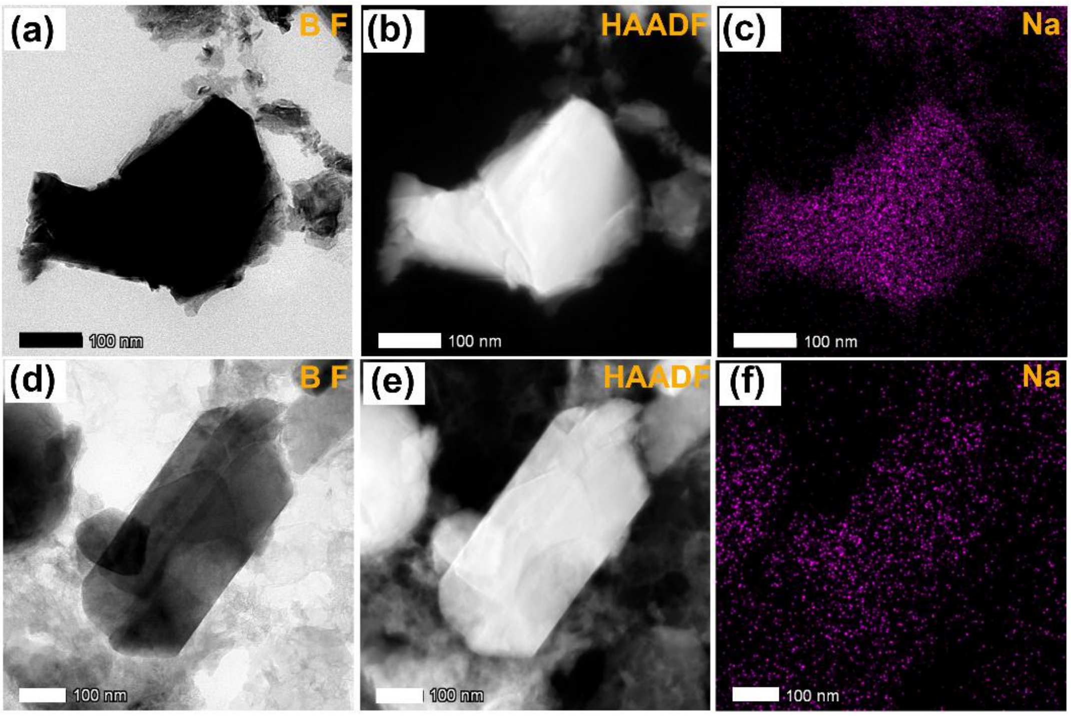

Figure S16. Morphology and TEM images of Na-LMR electrode primary particles before (a-b) and after (d-e) 200 cycles at 0.5C rate in BF mode and HAADF mode. (c) the uneven distribution and segregation of Na^+ in Na-LMR electrode before cycling and (f) in Na-LMR electrode after 200 cycles.

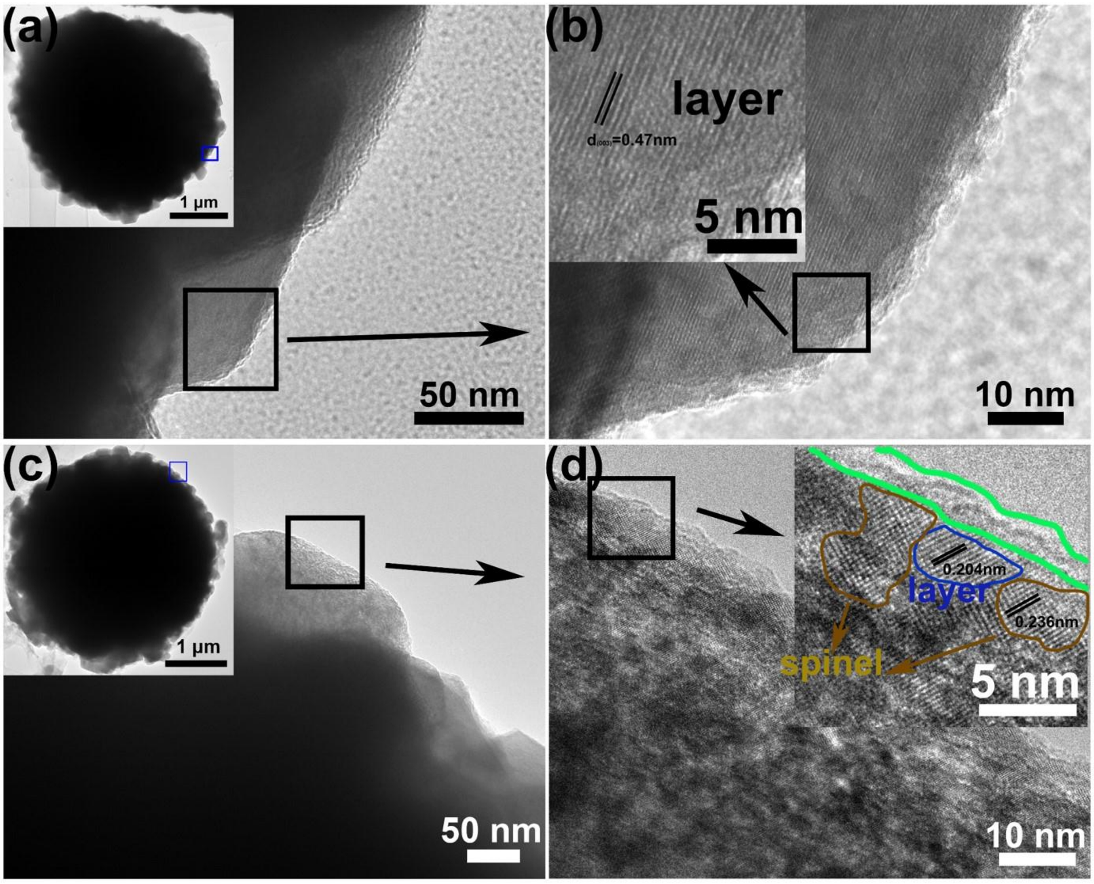

Figure S17. The HRTEM images of Na/SDS-LMR materials after 200 cycles at 0.5C rate and the inserted secondary particle (a); the corresponding primary particle (b), the inner fringes with a lattice spacing of 0.47 nm confirm the (003) plane of well-defined layered phase; the HRTEM images of Na-LMR materials after 200 cycles at 0.5C rate and the inserted secondary particle (c); and the corresponding primary particle (d), the lattice spacing of 0.204 nm is confirmed as the (104) plane of layered-phase and 0.236 nm is confirmed as the (222) plane of spinel structure. The Na/SDS-LMR materials still kept an integrated layer structure after long cycles, however the Na-LMR suffered a terrible corrosion, there are spinel phase presenting to the electrodes which is consistent with the analysis of primary particles in Fig. 4.
